# Complete mitochondrial genome of *Gazella subgutturosa reginae* (Bovidae: Antilopinae)

**DOI:** 10.1080/23802359.2021.1950056

**Published:** 2021-07-09

**Authors:** Wen Qin, Jirong Li, Changhui Xu, Le Yang

**Affiliations:** a State Key Laboratory of Plateau Ecology and Agriculture, Qinghai University, Xining, China; b Institute of Agricultural Product Quality Standard and Testing Research, Tibet Academy of Agricultural and Animal Husbandry Sciences, Lhasa, China; c Animal Husbandry Station of Leiwuqi County, Changdu, China; d Tibet Plateau Institute of Biology, Lhasa, China

**Keywords:** Mitochondrial genome, Qaidam basin

## Abstract

In Qinghai province, *Gazella subgutturosa reginae* (Adlerberg, 1931) is only distributed in Qaidam basin and it is beneficial for the balance of this ecosystem. In this paper, we present the complete mitochondrial genome of *Gazella subgutturosa reginae* firstly, a circularized sequence with 16,435 bp, containing a total of 13 protein coding genes, 22 transfer RNA (tRNA) genes, and 2 ribosomal RNA (rRNA) genes. The sequence is similar to other subspecies of *Gazella subgutturosa*, the phylogenetic tree revealed that *Gazella subgutturosa reginae* and *Gazella subgutturosa subgutturosa* are more closely related to each other. Our research is useful for the taxonomic and evolutionary research of goitered gazelle.

*Gazella subgutturosa* is also known as goitered gazelle because the male’s larynx is swollen like the head of a gander during rut and they prefer dry and desert environments (Xu et al. 2008). At present, six subspecies have been reported around the world, the subspecies in Qaidam basin is *Gazella subgutturosa reginae* (Sun et al. 2002). Goitered gazelle is one of the most important herbivores in Qaidam basin, and they are beneficial for the balance of ecosystem. Unfortunately, little is known about *Gazella subgutturosa reginae.* For a deeper knowledge of this species, here we reported the sequences of complete mitochondrial genome of *Gazella subgutturosa reginae* firstly (GenBank accession number: MW285638). The raw data accession number is PRJNA680197 in Genbank.

The sample was collected from a dried skin in Dulan county of Qaidam basin (Zongjia County, N36.3715° E96.7662°) on 31 July 2016. Genome DNA was extracted with HiPure Universal DNA Kit (Magen, Guangdong. China) and paired-end sequenced (2 × 150) on an Novaseq 6000 platform (Illumina, San Diego. USA), NOVOplasty 4.0 (Dierckxsens et al. 2017) was used for mitochondrial genomes reconstruction (type = mito; Genome Range = 12,000–22,000; K-mer = 33), then MitoZ software (Meng et al. 2019) was used for reordering the circularized sequence with NC_020710.1 as a reference (Hassanin et al. 2012), and annotation was performed with default parameters.

We obtained a circularized sequence with a length of 16,435 bp, it is similar to other subspecies of goitered gazelle, containing a total of 13 protein coding genes, 22 transfer RNA (tRNA) genes and 2 ribosomal RNA (rRNA) genes. The phylogenetic tree was built based on 46 complete mitochondrial genome, including 45 species and subspecies from Antilopinae and *Bos mutus* as the outgroup, all the sequences except *Gazella subgutturosa reginae* were downloaded from Genbank. Meanwhile, we only chose one species from each genus, however, there are 10 species and subspecies from gazella. All sequences were assembled with MAFFT (–maxiterate 1000 –globalpair –thread 256) (Katoh and Standley 2013). We used ModelFinder to find the Best-fit model; based on BIC, it is GTR + F+R5 (Kalyaanamoorthy et al. 2017). A maximum likelihood tree was built using IQ-TREE with analysis type: ModelFinder + tree reconstruction + non-parametric bootstrap (1000 replicates) (Minh et al. 2020) (Figure 1). At last, the tree was shown using ITOL (Letunic and Bork 2021). We re-rooted the tree according to the outgroup.

**Figure 1.**
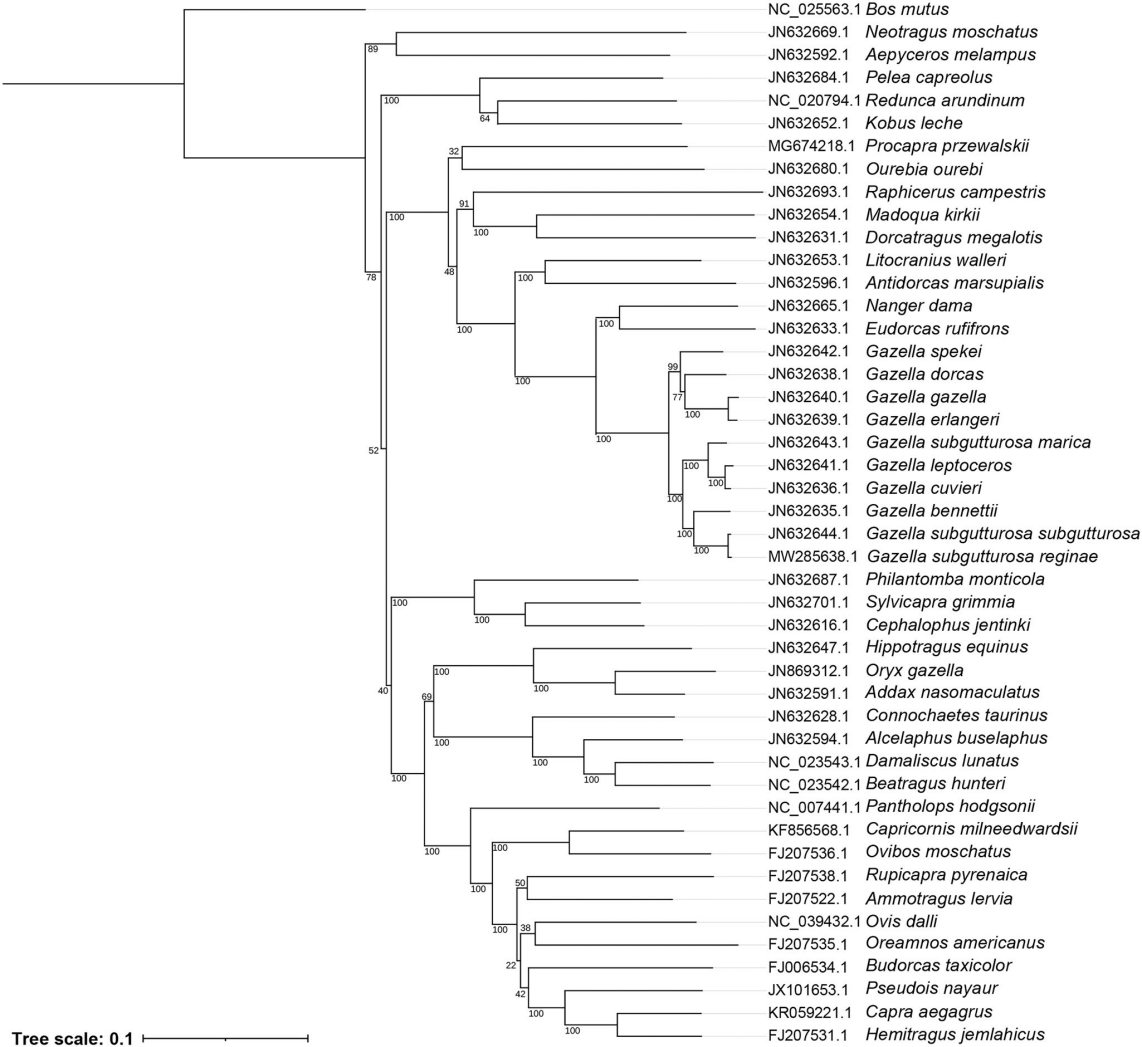
Maximum likelihood tree of subfamily Antilopinae including 45 species and subspecies with *Bos mutus* as the outgroup. The number around each node indicates the maximum likelihood tree bootstrap support values.

The phylogenetic tree shows a closer relationship between *Gazella subgutturosa reginae* and *gazella subgutturosa subgutturosa*; however, the relationship between *Gazella subgutturosa reginae* and *Gazella subgutturosa marica* is opposite. We speculate that this is caused by geographical distance. As *Gazella subgutturosa reginae* was collected in China, it is close to Turkmenistan where *gazella subgutturosa subgutturosa* was found, and Arabian Peninsula, where *Gazella subgutturosa marica* was found; it is relatively far away, but more evidence is needed (Hassanin et al. 2012). Our results are useful for the taxonomic and evolutionary research of goitered gazelle.

## Data Availability

The data that support the findings of this study are openly available in GenBank of National Center for Biotechnology Information at https://www.ncbi.nlm.nih.gov, reference number MW285638 and PRJNA680197. A specimen was deposited at Key Laboratory of Adaptation and Evolution of Plateau Biota, Northwest Institute of Plateau Biology, Chinese Academy of Sciences (contact Dr. Zhenyuan Cai email: caizhenyuan@nwipb.cas.cn) under the voucher number GPS 669.
